# Mopane worm value chain in Zimbabwe: Evidence on knowledge, practices, and processes in Gwanda District

**DOI:** 10.1371/journal.pone.0278230

**Published:** 2022-12-05

**Authors:** Faith A. Manditsera, Juliet Mubaiwa, Tonderayi M. Matsungo, Prosper Chopera, Sandra Bhatasara, George Kembo, Honest Mahlatini, Faith Matiza Ruzengwe, Felix Matutu, John Grigor, Alberto Fiore, Lesley Macheka

**Affiliations:** 1 Department of Food Science and Technology, Chinhoyi University of Technology, Chinhoyi, Zimbabwe; 2 University of Zimbabwe, Mt Pleasant, Harare, Zimbabwe; 3 Food and Nutrition Council, Harare, Zimbabwe; 4 Forestry Commission of Zimbabwe, Gwanda, Zimbabwe; 5 Division of Engineering and Food Science, Abertay University, Dundee, United Kingdom; 6 Marondera University of Agriculture Science and Technology, Marondera, Zimbabwe; International Maize and Wheat Improvement Centre: Centro Internacional de Mejoramiento de Maiz y Trigo, MEXICO

## Abstract

Consumption of edible insects is a potential solution to the growing need for protein. However, the wild harvested edible insects’ value chain faces several challenges including limited knowledge on indigenous practices in the harvesting and processing and lack of information on roles of the different actors in the chain. A case study of *Gonimbrasia belina*, colloquially referred to as the ’mopane worm’, was conducted to understand and identify determinants of participation in the value chain of the edible caterpillar. A cross sectional study was conducted in Gwanda (a rural district in Zimbabwe) to (a) understand the indigenous knowledge on harvesting and processing methods, (b) explore value addition and the traditional beliefs surrounding the utilisation of the mopane worm. Results showed that consumers (81.7%), and harvesters (76.6%) were the main actors in the mopane worm value chain. Using the Ordinary Least Squares (OLS) model, the following were shown to be determinants of participation in the mopane worm value chain: (a) gender (b) household size (c) marital status (d) religion and (e) household assets. Two primary processing methods of harvested mopane worm were distinguished i.e., boiling and roasting on ambers. Results showed lack of diversity in mopane worm-based products. Current culturally acceptable processing techniques need improvement and standardization to support sustainable mopane worm processing while optimising nutrient bio-accessibility.

## 1. Introduction

World population, which is projected to reach 9 billion in 2050, requires new and innovative methods of producing food to be identified and up-scaled [1]. Apart from preventing any major shortfall in global food supply, these new food production systems should be sustainable, i.e., culturally acceptable, accessible, economically fair and affordable, and able to provide adequate diets [2]. In particular, with this increasing human population and subsequent environmental degradation, the world faces a major problem in providing adequate animal-based protein. The consumption of edible insects, also referred to as entomophagy, is viewed as a potential part of the solution to the growing need of animal-based protein [3, 4]. The FAO identified edible insects as a sustainable alternative to close this anticipated food deficit, particularly protein, because insects are highly nutritious and have a low carbon footprint [5]. This increasing recognition and importance of edible insects to human nutrition has been supported by several studies [6–8]. Therefore, nutritionally, edible insects are a rich source of protein and minerals (e.g. iron, calcium and zinc) [9, 10] which could be part of a range of mitigating strategies to reduce the impact of population growth and climate change on food insecurity, which is being faced in some parts of the world already.

Consumption of edible insects is already a common practice in Zimbabwe and the world at large, where it plays an important role as a diet supplement [7, 11]. Larvae of the emperor moth, *Gonimbrasia belina*, colloquially referred to as ‘mopane worm’ is the most consumed [12, 13] and commercialised insect in Zimbabwe and its value chain is well advanced as compared to value chains of other non-timber forest food products [14]. Nutritionally, mopane worms represent a cheap protein-rich source that can improve the food and nutrition security status of rural households [13]. Biochemical analysis indicates that the insect is rich in protein content (approx. 58–65% DM) [15], fat content (approx. 15% DM of which 38% fatty acids are saturated and 62% are unsaturated), carbohydrates (approx. 8% DM) and considerable proportions of minerals (approx. 1.33% DM) [16, 17]. However, mopane worm also contains high levels of lysine, tryptophan, and methionine and a considerable amount of antinutritional factors such as chitin [18]. Freshly harvested and dry mopane worms are consumed in many ways after processing. Usually, the mopane worms are consumed as a relish with thick porridge. However, due to the texture of mopane worms, young children cannot consume them, hence are not able to benefit from the high nutrients in mopane worms. According to the 2020 Zimbabwe Vulnerability Assessment Committee (ZimVAC) livelihoods assessment report [19], mopane worm is mainly consumed in Matabeleland South and North provinces. The ZimVAC report further revealed that 83% of the surveyed population in Matabeleland South and 76% in Matabeleland North provinces, indicated that they consume mopane worms.

A study by Kozanayi [20] to identify and characterise the mopane worm supply and distribution chains from harvest to consumption showed that collection and marketing of the mopane worm is a widespread and diverse activity that is usually carried out by the poor. Also, a high degree of opportunism and low barriers to entry characterized the trading of the mopane worm. However, the expansion of the mopane worm value chain faces several challenges including limited documentation of knowledge on indigenous practices in the harvesting and processing of the mopane worms, lack of information on roles of the different actors in the chain and demographics of households involved in the value chain. Moreover, the value chain is embedded in indigenous and cultural practices, confirming that food is culturally constructed [21]. There is also a long historical relationship between insects and human culture. However in most instances, indigenous and cultural practices are regarded as an aberration [22], therefore this study shows the significance of indigenous and cultural practices and, how these form the basis for any external interventions and up-scaling.

Therefore, this study was conducted to understand the indigenous knowledge on mopane worm harvesting and processing methods, value addition and the traditional beliefs surrounding the utilisation of mopane worm. Specific research questions were: (i) What are the indigenous harvesting and processing methods used in the mopane worm value chain? and ii) What are the value-added products and the traditional beliefs surrounding the utilisation of the mopane worm?

The significance of this study is in that the mopane worm value chain is a main source of income for many households in Gwanda District [17], yet this value chain is still underdeveloped [16]. This study therefore gives insights into the mopane worm value chain, the actors involved and identifies the available opportunities and barriers in participating in this value chain. Such information is key to understand opportunities for improvement and how to make the value chain more sustainable since livelihoods of many households in Gwanda district revolve around mopane worms. In addition, sustainability issues have been raised in this value chain [14, 23], mainly due to overharvesting [24] and lack of policies regulating harvesting [25], however so far there are still policy gaps. Few, if any attempts have been made to build on local values and beliefs on environmental conservation to support sustainable harvesting practices using appropriate policy tools. Therefore, findings from this study will help policy makers identify priorities in the mopane worm value chain.

## 2. Research methodology

### 2.1 Setting and study design

The study was conducted in a mopane woodland zone of Gwanda a rural district in Zimbabwe. Gwanda District was selected as the study site based on several considerations, particularly, the abundance of mopane worms in the district [17] and the general conditions of rural development in southern Africa characterized by high levels of poverty and diversified livelihoods.

A cross-sectional mixed methods design was used for the purposes of concurrent triangulation. Quantitative data were collected through an interviewer administered structured questionnaire (S1 File) and qualitative data through focus group discussions (FGDs).

### 2.2 Sampling and sample size determination

Sample size for the quantitative component was calculated based on availability of mopane worms using Dobson formular [26]. Information on availability of mopane worms was extracted from ZimVAC 2020 survey [19]. The availability and consumption of mopane worm guided the purposive selection of 4 administrative wards (Ward 14, 16, 21, and 22). Four enumeration areas (EAs) from each ward were randomly selected for data collection and households were identified using systematic random sampling from the list provided in the village registers by first selecting the first household at random using the lottery method. This was followed by selecting subsequent households guided by a sampling interval dependent on the proportionate households determined by the number of households in the village registers. In each EA, 19 households were selected to make up a total of 304 households. A household was defined as all members, related or unrelated, who share the same dwelling unit and resources such as meals.

### 2.3 Data collection and tools

The structured questionnaire (household interview) collected data on the edible insects’ value chain in general and that of mopane worm value chain in particular. In addition, information on harvesting, processing, consumption, and trading of mopane worm was also collected. Focus group discussions were conducted to get detailed information on knowledge, attitudes and practices (KAP) on mopane worm value chain. A structured questionnaire was developed based on the objectives of the survey and consisted of the sections shown in Table 1. Prior to the pilot phase, the applicability of the methodology and questionnaire design was discussed in a workshop with local researchers and government forestry officials who had extensive knowledge of the relevant biological, social, economic, marketing and institutional issues. This pilot phase led to revisions and amendments that determined the final experimental design.

**Table 1:** Type of data collected from FGD and structured questionnaire.

Activity	Description
**Introduction**	Introduction to the survey and consent
**Questionnaire identification**	Ward, enumeration area, enumerator, date
**Socio-demographic characteristics**	Age, level of education, source of income, household characteristics
**Indigenous knowledge**	Forms of knowledge, attitudes and practices on edible insects
**Value chain of edible insects**	Preferred edible insects, general consumer information on frequency and reasons for consumption of edible insects, preferred attributes of edible insects, preferred harvesting season, common harvesting methods, preferred harvesting time, challenges and opportunities.
**Mopane worm value chain**	General consumer information on frequency and reasons for consumption of mopane worms, preferred attributes, harvesting, processing, allergic reactions, trade.

The sample size for the FGDs was between 6 and 15 respondents. One FGD was carried out in each of the four selected wards. In each ward, an enumeration area was randomly selected for having the FGDs. The selected enumerations areas for FGDs were: Bhalula (Ward 14), Riverblock (Ward 22), Mafume village (Ward 16) and Georgia farm (Ward 21). The FGDs constituted adult males, adult females, youth, harvesters, processors, traders, consumers, and other community members engaged in mopane worm value chain. Discussions were conducted in the local language that was best understood by all informants and later transcribed. World Health Organisation Protocols for prevention of Covid-19 procedures and guidelines for conducting surveys and group discussions (i.e., social distancing, hand sanitizing and wearing masks) were followed to ensure safety of participants and enumerators.

### 2.4 Data handling and statistical analyses

Primary household data were transcribed using CSEntry (CSPro) on android gadgets. A total of 304 questionnaires were administered by enumerators who were drawn from the database of enumerators previously engaged in ZimVAC surveys and had experience of collecting data using android devices.

#### 2.4.1 Quantitative data analysis

Household data transcribed using CSEntry (CSPro) on android gadgets were consolidated and converted into IBM SPSS version 25 and Stata version 14 datasets for analysis. Chi-square analysis was used to determine whether there were statistically significant differences between participators and non-participators with regards to gender, among ethnicity, marital status, religion, education, family member who participated and source of income. Statistical significance was tested at the 5% level. Regression analysis was used to determine the household characteristics and outcomes associated with participation in the mopane worm value chain such that;

$$\text{Pr}\;\left(\text{Participation}=1|\text{X}\right)=\text{f}\left(\text{X}\right)=\beta_{0}+\text{X}’\;\beta_{1-\text{k}}.$$ (1)

where the variable *Participation* takes the value of 1 if the household participates in mopane worm value chain and 0 otherwise, f(X) is logistic distribution function and **X** is a vector of control variables. β_0_ is a constant and β_1-k_. is a (1 x k) scalar of control coefficients.

#### 2.4.2 Qualitative data analysis

Qualitative data analysis was multi-layered and primarily inductive to allow themes, issues, trends, patterns, and conclusions to emerge from the data. Due to the intuitive and inductive nature of qualitative data, thematic analysis consisted of three specific activities, (i) scrutinizing the data for themes, concepts and propositions, (ii) manually coding the data and refining what one understands of the perceptions provided by research participants, and (iii) searching for recurring themes across all the study sites. These recurring themes were the basis for arguments and conclusions made in this paper.

### 2.5 Ethics and approvals

Ethical clearance was obtained from the Research Ethics Committee at Marondera University of Agricultural Science and Technology (MUAST12/20). Community leaders and government officials from Gwanda district were consulted before meeting household representatives to obtain verbal informed consent after explanation of research intentions and protocols. Respondents were briefed on overview of the study and an informed consent was obtained before commencement of data collection (see S1-Questionnaire page 1 for consent in S1 File).

## 3. Results and discussion

### 3.1 Characteristics of participating and non-participating households

Mopane worms were the most consumed (74.8%) edible insects in the surveyed households mainly because they are readily available in the district. Another commonly consumed insect was the termite (*Macrotermes* spp.) (6.5%). These results support previous findings by Dube [11], which showed that mopane worm (90%) and termites (80%) were the most consumed edible insects in Zimbabwe. Our study showed that at least 66.7% of the surveyed households indicated that they participated in the mopane worm value chain. Some of the households were involved in more than one stage of the value chain.

Table 2 shows the characteristics of surveyed households in relation to their participation in the mopane worm value chain. The results reveal that there was no significant association between participation in the mopane worm value chain and gender (*χ*^*2*^ = 14.177; df = 8; p = 0.077). However, a significant association was found between participation in the mopane worm value chain and source of income (*χ*^*2*^ = 75.025; df = 28; p = 0.000), religion (*χ*^*2*^ = 60.294; df = 24; p = 0.000), ethnicity (χ^*2*^ = 48.952; df = 20; p = 0.000), marital status (*χ*^*2*^ = 63.898; df = 20; p = 0.000), and education level (*χ*^*2*^ = 107.303; df = 24; p = 0.000).

**Table 2:** Household characteristics by participation in mopane worm value chain status^1^.

Variable	Harvester	Processor	Trader	Consumer	P value
**Gender**					
Male	148 (50.7)	103 (50.7)	113 (50.7)	152 (49.8)	0.077
Female	144 (49.3)	100 (49.3)	110 (49.3)	153 (49.8)	
Total	292	203	223	305	
**Source of income**					
Formal employment	14 (25.5)	10 (18.2)	12 (21.8)	19 (34.5)	0.000
Informal employment	12 (24)	12 (24)	12 (24)	13 (27)	
Artisinal mining	14 (29)	12 (24)	10 (20)	13 (27)	
Casual labour	14 (25)	12 (21)	14 (25)	16 (29)	
Remittances	12 (25)	11 (23)	9 (19)	16 (33)	
Petty trade	12 (35)	7 (21)	7 (21)	8 (24)	
Farming	55 (31)	25 (14)	37 (21)	58 (33)	
Other	13 (28)	11 (24)	10 (22)	12 (26)	
**Religion**					
Protestant	19 (23)	13 (16)	17 (21)	32 (40)	0.000
Pentecostal	56 (33)	33 (19)	36 (21)	47 (27)	
Apostolic Sect	35 (24)	31 (22)	35 (24)	42 (29)	
Zion	33 (28)	22 (19)	28 (24)	33 (28)	
No religion	35 (26)	29 (21)	30 (22)	42 (23)	
Other	17 (31)	13 (24)	9 (16)	16 (29)	
**Ethnicity**					
Shona	21 (24)	18 (20)	23 (26)	27 (30)	0.000
Ndebele	120 (29)	85 (20)	94 (22)	121 (29)	
Sotho	58 (26)	42 (19)	49 (22)	74 (33)	
Other	24 (27)	18 (20)	19 (21)	29 (32)	
**Marital Status**					
Single/Never married	91 (27)	71 (21)	77 (23)	103 (30)	0.000
Married	128 (29)	87 (20)	94 (21)	129 (29)	
Divorced	12 (22)	9 (16)	13 (24)	21 (38)	
Widow/Widower	20 (25)	14 (18)	18 (23)	27 (34)	
Not Applicable	84 (30)	55 (20)	62 (22)	80 (28)	
**Education Status**					
No education	83 (23)	69 (25)	78 (27)	108 (26)	0.000
Primary	121 (33)	83 (31)	92 (31)	124 (30)	
ZJC	55 (15)	47 (17)	48 (16)	64 (15)	
Ordinary Level	88 (3)	56 (4)	60 (20)	87 (21)	
Advanced Level and Post level	12 (3)	11 (4)	10 (3)	21 (5)	
Other	9 (2)	6 (2)	5 (2)	9 (2)	

^1^ All values represent n (%), total n = 304 households except for multiple response questions.

* p value for Pearson’s Chi Squared test.

*p value was significant at p<0.05.

Regarding the main source of income for the participants at each stage of the mopane worm value chain, the highest proportion of harvesters relied on farming (31%) whilst for traders it was casual labour (25%), and a majority of the consumers were formally employed (34.5%). These findings confirm results from the ZimVAC (2020) rural livelihoods survey, which revealed that farming (24%) is the main source of income for rural households, followed by casual labour (22%). In addition, the finding that the main consumers of mopane worms are the formally employed indicates that the low status given to edible insect’s consumption as a poor man’s food is not correct. This finding corroborates findings from a survey by Stack [27] in which the author concluded that widespread collection of mopane worms is an indication that utilisation of this forestry resource is not limited to the poorest households but is an activity undertaken by all social classes.

The results further reveal that the highest proportion of harvesters and processors were Pentecostal (33%) and consumers were Protestant (40%). As for the ethnicity of the participants in the various stages of the value chain participants of Ndebele origin (29%) dominated the harvesting and processing stages, while participants of Sotho origin dominated the trade and consumption stages. This result reflects the ethnicity of the population of Gwanda district, where the study was conducted, which is dominated by the Ndebele people followed by the Sotho people [28]. In addition, the results show that married participants (35.9%) dominated the mopane worm value chain followed by the single/never married participants (26.6%). The results further show all stages of the mopane worm value chain were dominated by participants who had attained only primary education. This result can be attributed to the fact that the educated members of the society migrate to the urban areas and neighbouring countries in search for jobs and greener pastures, leaving the lowly educated members behind in the rural areas [29–31]. Advancement in education tend to increase the opportunities for gainful employment and therefore improved household income and welfare [31].

From the FGDs, participants noted that some churches barred congregants from consuming mopane worms hence this was one of the main reasons for non-consumption by some people in the area. For instance, the doctrine of the Zion church prohibits members from harvesting, consuming and trading in edible insects, although some members do not adhere to this rule. This finding is corroborated by Stack [27] who reported that some households not involved in collecting mopane worms belong to religious groups that forbid collecting of such products. More so, studies by Hlongwane [32], Lucchese-Cheung [33], Rovai [34], and Verbeke [35], revealed religion as one of the dietary restrictions against consumption of edible insects.

### 3.2 Determinants of household participation in the mopane worm value chain

Table 3 shows results of the Ordinary Least Squares (OLS), Logit and Probit regression models used to determine the background characteristics that influenced participation in the mopane worm value chain. Using the OLS model, the following were shown to be the determinants of participation in the mopane worm value chain: (a) gender (b) household size (c) marital status (d) religion and (e) household assets. The results show that there is a 16.8% likelihood for female headed household to participate in the mopane worm value chain. Increasing the household size by one-member is likely to result in 2.3% probability of the household participating in the mopane worm value chain at 5% level of significance. Baiyegunhi, Oppong [31] reported similar findings in a study on commercialisation of mopane worm in rural households in Limpopo Province, South Africa. The authors attributed the high participation of female headed households in mopane worm value chain to the lack of resources and access to productive assets (land, labour, capital) which limits their agricultural production capabilities, resulting in these households relying more on forest products for subsistence and cash income. Furthermore, the results indicate a 21.7% probability that members of the apostolic sect will not participate in the mopane worm value chain. Similarly, members of the Zion are 20.9% likely not to participate in the mopane worm value chain. Zion church and Apostolic church have similar doctrines which prohibit their members to consume edible insects and/participating in either harvesting or trading.

**Table 3:** Determinants of involvement in mopane worm value chain.

	(1)	(2)	(3)
VARIABLES	(OLS)Household involvement	(Logit)Household involvement	Probit (Household involvement
Household head age	-0.00128	-0.00433	-0.00682
	(0.00234)	(0.00670)	(0.0115)
Household head female	0.168**	0.532**	0.877**
	(0.0753)	(0.242)	(0.409)
Household size	0.0233**	0.0764**	0.126**
	(0.00933)	(0.0326)	(0.0541)
Ethnicity Ndebele	0.104	0.291	0.490
	(0.167)	(0.448)	(0.829)
Ethnicity Jahunda	0.958***		
	(0.206)		
Ethnicity Sotho	0.102	0.301	0.503
	(0.176)	(0.480)	(0.883)
Ethnicity Other	0.258	1.039*	1.695*
	(0.175)	(0.553)	(1.010)
Married	0.310***	0.904***	1.492***
	(0.116)	(0.329)	(0.553)
Divorced	0.197	0.552	0.907
	(0.180)	(0.508)	(0.827)
Widow/widower	0.0547	0.109	0.169
	(0.150)	(0.411)	(0.684)
Roman catholic	-0.0620	-0.193	-0.340
	(0.212)	(0.576)	(0.899)
Protestants	0.113	0.392	0.725
	(0.127)	(0.415)	(0.761)
Pentecostal	-0.0280	-0.0788	-0.149
	(0.0946)	(0.290)	(0.498)
Apostolic	-0.217**	-0.706**	-1.166**
	(0.105)	(0.311)	(0.527)
Other Christian	-0.202	-0.651	-1.052
	(0.159)	(0.438)	(0.744)
Zion	-0.209**	-0.626**	-1.052**
	(0.101)	(0.303)	(0.513)
African traditional religion	-0.0585	-0.0805	-0.111
	(0.317)	(0.968)	(1.752)
Primary level	0.0846	0.253	0.450
	(0.0908)	(0.274)	(0.463)
ZJC	-0.0762	-0.242	-0.390
	(0.124)	(0.351)	(0.597)
O’ level	0.00170	-0.0350	-0.0269
	(0.104)	(0.301)	(0.506)
A’ level	0.202		
	(0.173)		
Diploma/Certificate after primary	0.284**		
	(0.141)		
Diploma/Certificate after secondary	-0.0627	-0.226	-0.418
	(0.211)	(0.606)	(0.993)
Graduate/post graduate	0.113	0.218	0.485
	(0.229)	(0.743)	(1.422)
Total household income	-0.0301	-0.0895	-0.154
	(0.0242)	(0.0757)	(0.128)
Household assets	0.0199*	0.0625*	0.0995*
	(0.0110)	(0.0321)	(0.0551)
Constant	0.391	-0.320	-0.480
	(0.299)	(0.906)	(1.563)
Observations	272	272	272
R-squared	0.167		

**Notes**: Robust standard errors in parentheses.

*** p<0.01,

** p<0.05,

* p<0.1.

### 3.3 Mopane worm value chain

#### 3.3.1 Harvesting and processing practices of mopane worms

The mopane worm value chain in rural Zimbabwe begins at household level and the chain involves harvesting of the mopane worm, followed by pre-treatment (which is primary processing), storage and secondary processing.

*3*.*3*.*1*.*1 Harvesting*. Table 4 shows that 73.4% of harvesters reported that they harvest for both consumption and trade, corroborating the findings of Thomas [36] that mopane worm is used both for trade and household consumption. Considering that the greater percentage of the actors are consumers, it is expected that a greater proportion of the harvesters for trade would also consume part of the harvested worms. Results from the focus group discussions revealed that in instances where religion or cultural beliefs do not support consumption of edible insects, some households participated in harvesting for trade only as a source of livelihood. The consumption of mopane worms is anticipated to have an effect on the overall food security as it provides nutrients and income to value chain participants [10, 37]. The harvesting of mopane worm offers an opportunity for participating communities to improve their livelihoods through trading. However, the erratic outbreaks and supply and high inter annual variability in production means that these products are not a very reliable income source [37].

**Table 4:** Quantitative data on harvesting.

	Percent (%)
***Reasons for harvesting mopane worms***	
Only for household consumption	18.6
Only for trade	7.9
For both household consumption and trade	73.4
***Who is involved in harvesting***	
Whole family	29.8
Mother only	22.0
Mother and children	15.7
Mother and father	11.5
Father only	9.9
Children only	4.7
Any household member	4.2
***Mopane worm harvest location***	
Within the ward	72.2
Outside the ward but within nearby wards	13.3
Outside the ward in distant wards	14.4
***Time of the day mopane worms are harvested***	
Morning	61.7
Afternoon	1.1
Evening	0.6
Anytime of the day	36.6

The bulk of the harvesting was done by either the whole family (29.8%), mothers only (22%), mother and children (15.7%) and in some households the mother and father (11.5%) agreeing with the findings of [15, 27] who reported on widespread involvement in mopane worm collection by all categories of households. However, discussions from the focus groups indicated that harvesting of mopane worm is not necessarily a collaborative task across all households. In a few instances, each household member, including children harvest their own mopane worm, but joint harvesting is also sometimes practiced. As also indicated by Stack [27], within the household it is of interest to understand the pattern of gender roles for different activities related to mopane worm value chain. According to Thomas [36] and Nantanga [18], mopane worm harvesting period coincides with the rain-fed crop production season [15], forcing households to weigh between allocating their labour between the two activities. Moreover, of concern is the involvement of children in the harvesting which illustrates the inescapable role of children in the labour force. In rural areas it is common for children to ‘help’ their parents with routine chores and ‘productive’ activities from an early age. However, it is also important to note that outbreaks of mopane worms coincide with the school holidays.

The study revealed that mopane worms were mainly harvested within the wards (72.2%) as (Table 4). However, it should be noted that these results are of the residents of the ward and number of harvesters could be more than the reported as outsiders also visit the mopane worm areas during mopane worm season for harvesting. This finding agrees with Thomas [36] who reported that in Namibia, harvesters of mopane worms include local people living in surrounding villages and outsiders who travel to harvesting sites. Problems that arise with harvesting of mopane worms were highlighted by Gondo [14], who reported that many rural communities face uncontrolled harvesting, especially by people not residing in the area. To curb this problem, some communities have a restrictive harvest period policy to conserve the environment. However, according to Akpalu and co-authors [13] restrictive harvest policy may not lead to sustainable harvesting of the worm. Due to uncontrolled harvesting or poor mopane worm season in some wards, the decline in mopane worm quantities can lead to some harvesters travelling to other wards to look for the mopane worms. Harvesting outside wards is mainly practised by households who would want to trade the mopane worm, otherwise for household consumption, harvesting is mainly within ward. It will be imperative to evaluate the different harvesting policies on sustainability of the mopane worm value chain and also their possible adoption by communities.

Mopane worms were mostly harvested in the morning as reported by 61.7% of the survey participants. At least 36.6% of households did not have a particular set time for harvesting mopane worms as they harvested at any time of the day. From the focus group discussions, it was highlighted that early morning harvesting gives the harvesters the opportunity to collect the fully matured mopane worms which would be ready to burrow into the soil. Harvesting of mopane worms is more desirable during the fifth instar. This is due to the minimal effort needed for degutting as the caterpillars have little to no plant matter in their digestive tracts and have reached maximum growth [15, 38]. According to the locals, these worms do not require any degutting and they are much tastier when cooked. Due to the “premium quality” of mopane worm, they are reserved for household consumption and in instances where they are sold, the price is usually higher than that of degutted mopane worms.

The survey indicated that most of the mopane worms are harvested during the February-May season (89.7%) as compared to the October-December season (10.3%). This is contrary to report by Gondo et al. [14], that the numbers of mopane worms present during the second outbreak (March-April) are less than those occurring during December-January. Moreover, Hope [37] reported erratic outbreaks of mopane worms which also affects the choice of preferred season. Additionally, it will be interesting to establish the effect of seasonality on mopane worm quality parameters. According to van Huis [39], an insect’s nutritional value is affected by the diet, season, developmental stage, sex, species, and growth environment.

Harvesting methods were common across the communities studied and are similar to the methods reported in Namibia by Thomas [36]. Two main methods emerged from the focus group discussions, which are: picking the worms from the trees and/or picking the worms from the ground (especially those that are about to burrow into the ground) agreeing with Stack [14]. Mopane worms were collected from both the ground and from trees, usually the 5th instar stage, and the last stage before pupation. Mopane worms collected from the ground, immediately prior to pupation generally had little undigested food in their guts and are easier to process as also reported by Kozanayi [20]. However, the respondents reported that most mopane worms are collected from the trees while still feeding and so must be processed thoroughly to remove all undigested material from their gut. According to Stack [27], in Zimbabwe, few collectors use gloves since these are unaffordable to most people.

During focus group discussions, it was emphasised that the harvesting moment must be appropriate or timely so as to catch the best quality of the mopane worm. This view agrees with the report by van Huis [39] about the importance of harvesting stage on the quality of the insect. The quality parameters of the mopane worm can include size, appearance and nutritional value.

*3*.*3*.*1*.*2 Trading*. The survey reveals that the majority (35.1%) of the harvested mopane worms are sold to informal markets (roadside and open markets) whilst a lesser percentage is sold to the formal markets (retail) (5.3%). Local trading of mopane worms for income generation [40] and exporting to neighbouring countries such as South Africa, Botswana, Zambia and the Democratic Republic of the Congo has been previously reported [14, 20].

The survey revealed the complexity of the mopane worm supply chain and the potential to improve the trading network. There is no common criteria like a grading system for measuring the quality of mopane worm in place besides the one given for manually degutted and naturally degutted and method of primary processing. According to the locals, two methods of primary processing exist i.e., a) roasting the degutted worms in hot ambers followed by sun-drying (b) boiling degutted worms followed by sun-drying. The price for these mopane worms were different owing to processing method, harvesting stage and time of the year. Our findings of high price of mopane worms during off season agree with Odongo [41] who reported that traders stored edible insects to be able to sell them during off season when demand and prices are high. Additionally, according to Makhado [15], the price of mopane worm was depended on the location i.e. it was more expensive in urban areas. Quality aspects considered for mopane worm include method of degutting, size etc. The absence of a stipulated grading system for mopane worm is worrying, therefore designing a standard grading system is yet another necessity to improve the value chain. Primary processing is a common practise applied to edible insects before trading. Odongo [41] reported that a majority of edible insects’ traders did some form of primary processing before selling them.

*3*.*3*.*1*.*3 Processing methods*. Processing methods highlighted during the focus group discussions include degutting, boiling, sun-drying, roasting. Information on processing techniques was either passed on from older family members or shared from women cooperatives. An important part of the processing of mopane worms is removing the innards (de-gutting). What was clear is that there is no one way of ensuring that the process is done. In Bhalula Ward, it was noted by focus group members that “…*Some immediately degut them after picking at site*, *then some transport them to their homesteads/processing site where they degut them*”. At Georgia farm it was reported that “…*mopane worm is degutted first then placed in hot charcoal*. *The mature caterpillars do not need degutting*”. It is important to note the effect of time taken to degut the mopane worm on quality. During processing it is important that only the gut contents are removed and that a “yellow substance,” (associated with the fat content) which consumers prefer because of its nutritional value and perceived taste, is not removed.

Two main primary processing methods were reiterated across all the wards visited, and these are: Drying by roasting on fire ambers and boiling in salted water and then drying as also reported by [27]. “*The difference between the fire roasted and boiled insects is that charcoal treated caterpillars have less “thorns” than the boiled ones*” said a focus group member in River Block (Ward 22).

Another aspect with processing using charcoal is that sometimes the worms are not degutted. However, it also emerged that processing worms using fire ambers was not sustainable, since, across all wards it was highlighted that it required two wheelbarrows of firewood to get one gallon of processed mopane worms. Those that are boiled, salted and dried were also considered cleaner. However, the roasting methods are preferred by harvesters who also trade as the method results in more voluminous product as such few mopane worms can easily fill the containers in which they are sold. Stack [27] also reported the practice of roasting mopane worms by some collectors in which they place mopane worms in a pit, cover with hot coals and allow the build-up of heat to expel the gut contents. The use of hot ambers has an implication on the availability of firewood and food safety aspects. The implication of firewood shortages means that this processing technique is unsustainable in Zimbabwe. Firewood constitutes 49% of the total energy used [42] with over 90% of rural and urban households depending on firewood energy as a result of countrywide power shortages experienced daily. Therefore, it is a concern that, while demand for firewood continues to grow in rural Zimbabwe, rapid land use and deforestation have reduced the supply of firewood. Processing of insects also causes formation of potentially toxic compounds. The reactions (such as maillard reactions) that occur during roasting of mopane worms can result in the production of toxic compounds like heterocyclic aromatic amines and acrylamide or furan [43, 44]. Given the uncontrolled roasting of mopane worms in hot ambers, there is a high likelihood of occurrence of maillard reaction metabolites and Polycyclic aromatic hydrocarbons (PAHs) in the products. In view of this, it is important to suggest alternative processing methods as reported by Ghazoul [45] of using dry-roasting drum that is presumed to be more productive at the same time evaluating the sustainability and safety aspects of different processing techniques.

**Fig 1:**
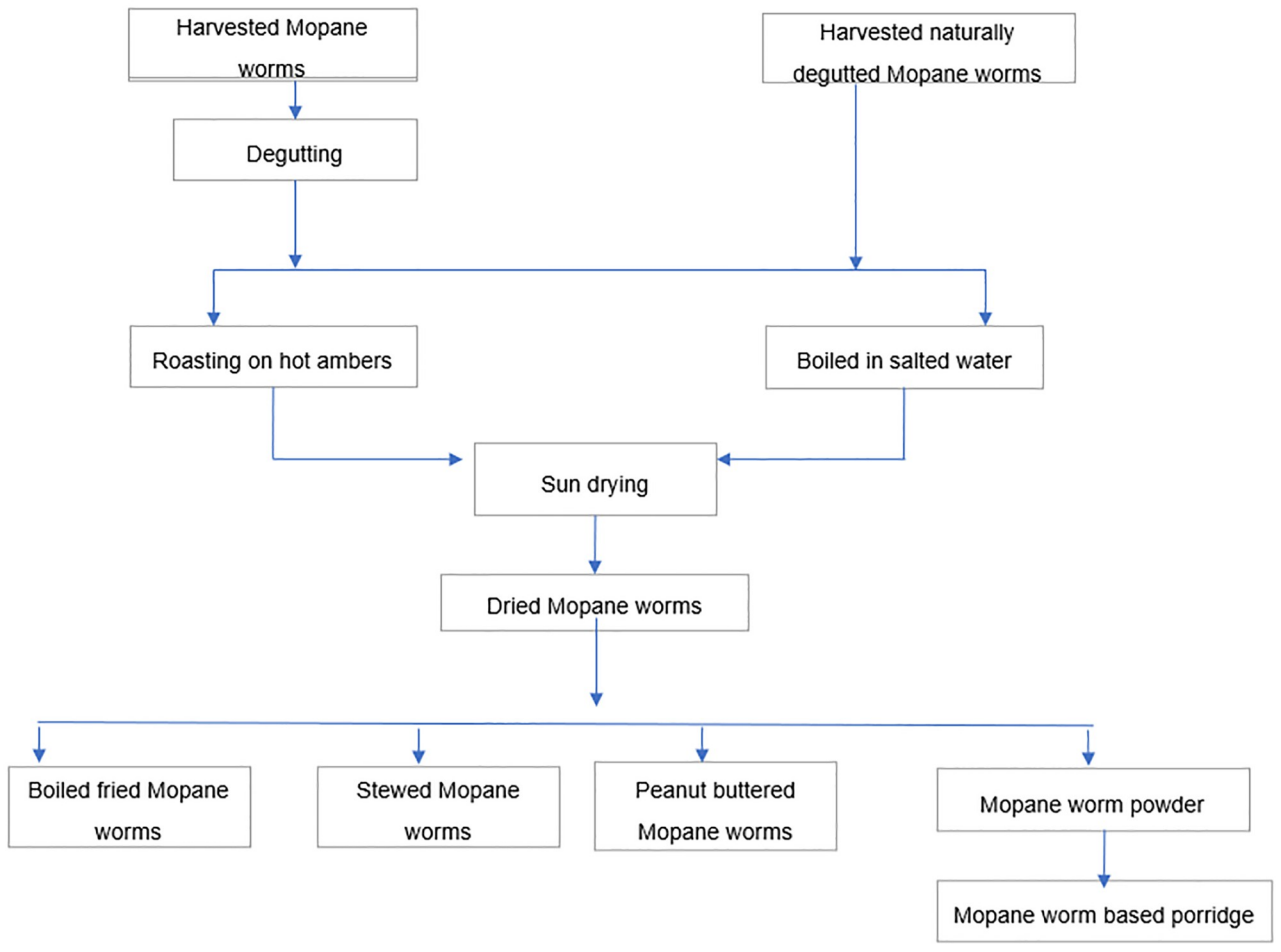
Mopane worm processing and product tree.

After degutting, mopane worms are washed with potable water. The washing step depends on the accessibility of water which will have an implication on the sustainability of the mopane worm value chain. According to the report by Stack [27] in Botswana and Zimbabwe, some processors skip this step if there is no water. People have various ways of cooking mopane worm as shown in Fig 1, but the most common are either boiling in salty water and then sun drying or roasting over a bed of hot coals followed by sun drying. The latter method is faster, uses less firewood and saves labour which is an advantage in the rainy season when a common problem is delay in drying due to wet and overcast days and shortage of firewood. Despite which method used, sun drying of mopane worms follows to achieve the final dry product and this process can take several days as the process is dependent of weather conditions. According to Manditsera [7] and Madibela [46], the steps of processing edible insects e.g. degutting, boiling and roasting can impact on the nutritional quality and bioaccessibility of proteins and minerals in the final product. In view of this, selecting applicable processing method is crucial to ensure the best nutritional and functional properties with good keeping quality of products [9, 10].

#### 3.3.2 Products derived from mopane worms

There was limited mopane worm products diversity in Gwanda district as shown in Fig 1. The most common way of consuming mopane worm is as a relish to thick maize porridge (*sadza)*. The only variations that exist are in the way the mopane worms are prepared as relish. Minimal value addition to marketed edible insects is common [41]. When asked to describe the products that are made from (or added to) the mopane worms before consumption, all participants said porridge. The specific recipes used varied from place to place, but the cooking process generally involved pounding the worms, sieving them to remove grit from skin and then incorporating the powder into a cereal-based porridge. Fortification of maize with inexpensive sources of plant proteins has been used as a strategy to help alleviate the ever-increasing problems of malnutrition in developing countries [47]. Blending of maize with a protein rich food has been reported as a strategy to improve the nutritive value of the staple which lacks some essential amino acids (lysine and tryptophan), minerals (such as calcium, potassium, iron and zinc) [47–49]. The quality of the porridge produced i.e., nutritional, functional and sensorial properties will depend on the pre-treatment of flour that is used in the formulation of the porridge. Different formulations of porridge were reported which included the use of different parts of the insects in blending with a cereal. Optimization of mopane worm/ cereal porridge is key in maximising bioaccessibility of important nutrients and contributing to the recommended daily allowance (RDA) of children and the rest of the household. However, successful performance of mopane worm flour as a food ingredient depends on the functional characteristics and sensory qualities it imparts to products. Functional properties are intrinsic physico-chemical characteristics that affect behaviour of foods during pre-treatment and storage, e.g., solubility, foamability, gelation and emulsification. To improve the use of mopane worm flour as a food ingredient, the functional properties and the characteristics imparted to food stuffs need to be assessed. Eventually, consumption of mopane worm can be stimulated by processing the insect into some flour, easing the utilisation and providing more diversity in local diets.

## 4. Conclusions and recommendations

The findings of the study show consumers, harvesters, traders, and processors as the participants of the mopane worm value chain and their involvement being determined by gender, household size, marital status, religion, and household assets. Large households and married people are more likely to participate in the mopane value chain than small size households and single persons respectively. Within the household it is of interest to understand the pattern of gender roles for different activities related to mopane worm value chain. Harvesting of mopane worm is mainly done for combined purpose of consuming and trading. Even though harvest of mopane worms offers an opportunity for income generation, unreliability of outbreaks necessitates innovation to ensure their constant supply. This means speeding research on semi-domestication and evaluating its impact on yield and quality parameters.

The survey showed degutting, boiling, roasting on ambers and drying as important processing techniques for mopane worms in Gwanda, but there was no clear cultural trend on processing diversity. The goal of mopane worm processing is not only to improve storage ability but to retain the safety, sensorial and nutritional attributes of the products. The mopane worm processing practices can be appraised from several perspectives, such as sustainability of the method, i.e., water and firewood, processing time, as well as the sensorial, safety and nutritional aspects of the final product. The water and firewood scarcity are important factors to consider in optimizing the mopane worm value chain as these can reduce the sustainability index of mopane worms. Moreover, to realise the full potential of mopane worm value chain, it’s imperative to evaluate the safety aspects of traditional processing techniques and their metabolites as this has an impact on adoption of this resource as food for the future.

Overall this necessitate investigating the correlation between processing technique and sustainability indicators to optimise current processing methods. Assessment of nutrient bioaccessibility and product functionality are necessary in evaluating the quality of the food products. As such, protein digestibility and micronutrient bioaccessibility studies are paramount as nutrient deficiency is still a problem that plagues sub-Saharan African communities compounded by the recent Covid-19 pandemic. Ultimately, knowledge sharing, education, building from existing strategies and applied research are essential interventions for improving food and nutrition security through insects.

## Supporting information

S1 File Survey questionnaire. (PDF)
