# Supplementary material for: Mopane worm value chain in Zimbabwe: Evidence on knowledge, practices, and processes in Gwanda District
Source: PLoS One. 2022 Dec 5;17(12):e0278230. doi: 10.1371/journal.pone.0278230 (PMC9721488; doi:10.1371/journal.pone.0278230)
Supplement: S1 File — (PDF) [file pone.0278230.s001.pdf]

Insects4Nutrition Project
Chinhoyi University of Technology, Marondera University of Agricultural Sciences and Technology, University of Zimbabwe, Food and Nutrition Council

Indigenous knowledge on edible insects and processing of insect-based porridge

Introduction statement

My name is ……………………………………… I am an enumerator for Insects4Nutrition project. We are conducting a survey on the "Indigenous knowledge on edible insects and processing of insect-based porridge''. This is part of a bigger project; whose purpose is to assess the contribution of insect-based porridge processing and consumption towards health and nutritional status of primary school children in Zimbabwean low socio-economic communities. The survey will gather information on the edible supply chain (harvesting, post-harvesting and end products) and use of edible insects in cereal based products. Your household is among several other randomly selected households to represent your ward and we would like to ask you few questions related to the project with the intention of finding ways to improve insect-based porridge processing and, consumption and subsequently nutrition security in Zimbabwe. The information to be collected is solely for research purposes. Your personal information will remain confidential and no responses will be linked to your identity. Would you like to participate in this survey?

Do you accept to be a participant in the survey?

Name……………………………………………………Yes/No

Date……………………………

QUESTIONNAIRE IDENTIFICATION

**Name of Province**		**Province Code**	
**District Name**		**District Code**	
**Ward Number**		**Household Number**	
**EA Number**		**Enumerator's Name**	
**Village Name/Farm Name**			
**Date of Interview (DD/MM/YYYY) e.g. 09/05/2020**			

1: HOUSEHOLD RESPONDENT CHARACTERISTICS

N°	Questions	Responses	Code
1.1	Respondent's Name		
1.2	Gender	1. Male<br>2. Female	
1.3	Age in completed years	1. ≤18<br>2. 19-29<br>3. 30-39<br>4. 40-49<br>5. 50-59<br>6. 60+	
1.4	Ethnic group	1. Shona<br>2. Ndebele<br>3. Jahunda<br>4. Sotho<br>5. Other (specify)...	
1.5	Marital status	1. Single/Never married<br>2. Married<br>3. Divorced<br>4. Widow/ widower	
1.6	Religion	1. Roman Catholic<br>2. Protestants<br>3. Pentecostal<br>4. Apostolic Sect	

		5. Other Christian<br>6. Islam<br>7. Zion<br>8. African traditional religion<br>9. No religion	
1.7	Highest level of education attained	1. No education<br>2. Primary level<br>3. ZJC<br>4. O-Level<br>5. A-Level<br>6. Certificate/Diploma after primary<br>7 Certificate/Diploma after secondary<br>8. Graduate/Post graduate<br>9. Other (specify)...	

2: SOCIO-DEMOGRAPHIC CHARACTERISTICS

N°	Questions	Responses	Code
2.1	NAME of household member		
2.2	Is respondent the household head?	1. Head of Household<br>2. Spouse<br>3. Son/Step Son<br>4. Daughter/Step Daughter<br>5. Uncle<br>6. Aunt<br>7. Nephew<br>8. Niece<br>9. Father/Father in Law<br>10. Mother/Mother in Law<br>11. Daughter in Law<br>12. Son in Law<br>13. Brother<br>14. Sister<br>15. Other relative	
2.3	Gender	1. Male<br>2. Female	
2.4	Ethnic group	1. Shona<br>2. Ndebele<br>3. Jahunda<br>4. Sotho<br>5. Other (specify)...	
2.5	Marital status	1. Single/Never married<br>2. Married	

		3. Divorced<br>4. Widow/ widower	
2.6	Religion	1. Roman Catholic<br>2. Protestants<br>3. Pentecostal<br>4. Apostolic Sect<br>5. Other Christian<br>6. Islam<br>7. Zion<br>8. African traditional religion<br>9. No religion	
2.7	Highest level of education attained	1. No education<br>2. Primary level<br>3. ZJC<br>4. O-Level<br>5. A-Level<br>6. Diploma/Certificate after primary<br>7 Diploma/Certificate after secondary<br>8. Graduate/Post graduate<br>9. Other (specify)..	
2.8	Age in completed years	1. ≤18<br>2. 19-29<br>3. 30-39<br>4. 40-49<br>5. 50-59<br>6. 60+	
2.9.1	Is NAME biological mother alive (If age is <17 years)?	1. Yes<br>2. No	
2.9.2	If alive does the biological mother live in this household(If age is <17 years)?	1. Yes<br>2. No	
2.9.3	Is %MEMBER_NAME% 's biological father alive (If age is <17 years)?	1. Yes<br>2. No	
2.9.4	If alive does the biological father live in this household (If age is <17 years)?	1. Yes<br>2. No	

3: SOCIO-ECONOMIC HOUSEHOLD CHARACTERISTICS

N°	Questions	Responses	Code
3.1	What is your household main livelihood source (source of income)?<br>*(possible to give multiple responses in order of importance)*	1. Formal employment<br>2. Informal employment<br>3. Commercial farming<br>4. Artisinal mining activities (makorokoza)<br>5. Casual labour<br>6. Remittances<br>7. Petty trade<br>8. Insect trading<br>9. Pension<br>10. Subsistence farming activities<br>11. Cross border trading<br>12. Other (specify)...	
3.2	What are your household's other sources of livelihood (other sources of income)?<br>*(multiple responses)*	1. Formal employment<br>2. Informal employment<br>3. Commercial farming<br>4. Artisinal mining activities (makorokoza)<br>5. Casual labour<br>6. Remittances<br>7. Petty trade<br>8. Insect trading<br>9. Pension<br>10. Subsistence farming activities<br>11. Cross border trading<br>12. Other<br>(specify)...	

4: HOUSEHOLD INCOME IN THE PAST CALENDER MONTH

**4.** What was the estimated total amount of income earned by your household from each of the following activities in the **last** calendar **month (March 2021)?**				
**Income Sources**	**Cash**		**In-kind**	
	**Amount**	**Currency** ***1= RTGS$ 2= USD 3 = Rand 4 = Pula 99=N/a***	**Equivalent Amount**	**Currency** ***1= RTGS$ 2= USD 3 = Rand 4 = Pula 99=N/a***
1. Opening balance as at 1 March 2021				
2. Can you estimate how much your household earned in CASH from all income sources during the month of March 2021 ?				
3. Can you estimate how much your household earned INKIND from all sources during the month of March 2021? (convert all INKIND items to CASH equivalent)				
4. Can you estimate how much this household averagely generates on a monthly bases from all sources				
5. Can your estimate how much your household earned [RANGE] from your household's main livelihood source (main income source) in last 12 months (1 April 2020 to 31 March 2021)?	**Responses**<br><br>1=0 to 500<br>2=501 to 1 000<br>3=1 001 to 5 000<br>4=5 001 to 10 000<br>5=10 001 to 20 000<br>6=20 001 to 50 000<br>7=50 001 to 100 000<br>8=Greater than 100 000			

5: HOUSEHOLD DIETARY DIVERSITY

5.1 How many meals **did the members in your household** aged 5 years and above eat **yesterday**? **(Number of meals)**					
5.2 Is this the usual number of meals **these members have in a day**?					**0 = No**<br>**1 = Yes**
5.3 Over the last **seven days**, how **many days** did your household consume the following food items/products and **what** was the **main source** of each food item? **H**ow these foods were acquired? (write 0 **for Main Sources if food item was not consumed)**					
		Number of days eaten in past **7 days**	Number of days eaten in past **7 days (combined)** If 0 move to next food item**)**		Did your household consume the following food items **yesterday (breakfast, lunch, dinner, drops)**?<br><br>*0= No*<br>*1= Yes*
1. Cereals and grain: rice, pasta, bread, sorghum, millet maize, maize meal, corn-soya blend, super cereal					
2. Roots and tubers: potato, yam, cassava, sweet potato, and/or other tubers					
3. Pulses	a. Sugar Beans				
	b. Other legumes/ nuts: cowpeas, peanuts, lentils, nut, soya beans, pigeon peas and/or other nuts				
4. Vegetables	a. Orange vegetables (vegetables rich in vitamin A): carrot, red pepper, pumpkin, butternut, orange sweet potatoes				
	b. Green leafy vegetables: spinach, broccoli, and/or other dark green leaves, cassava leaves				
	c. Other vegetables: onion, tomatoes, cucumber, green beans, peas, lettuce				
5. Fruits	a. Orange fleshed fruits (fruits rich in Vitamin A): mango,				

	apricot, peach, paw-paw **<u>excluding citrus fruits</u>**				
	b. Other fruits: banana, apple, lemon, naartjies, oranges, avocado				
6. Meat	a. Beef, goat, pork, game				
	b. Poultry				
	c. Liver, kidney, heart and/or other organ meats (*excluding tripe, cassings)*				
	d. Fish: fresh fish, sun-dried fish, and/or canned fish (fish in large quantities not as a condiment)				
7. Eggs					
8. Milk and other dairy products: fresh milk/ sour, yoghurt, cheese, other dairy products **(exclude margarine, butter or small amounts for tea/coffee)**					
9. Oil/ fat/ butter: vegetable oil, palm oil, butter, margarine, other fats/ oil					
10. Sugar, or sweet: sugar, honey, jam, candy, cookies, pastries, cakes and other sweet sugary drinks					
11. Condiments/ spices: tea, coffee, cocoa, salt, garlic, spices, yeast/ baking powder, tomato sauce, meat or fish as a condiment,					
12. Edible insects?					

6: DIETARY DIVERSITY FOR SCHOOL GOING CHILDREN 7-11YRS

N°	Questions	Responses	Code
6.1	**Are there children aged** 7-11 years currently living in the household?	0 = No 1 = Yes	
**If yes, yesterday during the day or at night, did the child eat or drink: (including those consumed outside the Household)**			
A.	Any foods made from grains, like: Porridge, bread, rice, pasta/noodles or other foods made from grains?	0 = No 1 = Yes	
B.	Any vegetables or roots that are orange or orange coloured inside, like: pumpkins, carrots, squash or sweet potatoes?	0 = No 1 = Yes	
C.	Any white roots and tubers such as white potatoes, white yams, cassava, or any other foods made from white-fleshed roots/tubers?	0 = No 1 = Yes	
D.	Any dark green leafy vegetables, such as: spinach, broccoli, kale, cassava leaves, pumpkin leaves, blackjack leaves?	0 = No 1 = Yes	
E.	Any fruits that are dark yellow or orange inside, like ripe mango and ripe paw-paw excluding citrus fruits?	0 = No 1 = Yes	
F.	Any other fruits like bananas, apples, lemon, oranges and avocado, including citrus fruits?	0 = No 1 = Yes	
**6.2 Yesterday during the day or at night, did the child eat or drink: (including those consumed outside the Household)**			
A.	Any other vegetables like onion, tomato, cucumber, green beans, peas and lettuce	0 = No 1 = Yes	
B.	Any meat made from animal organs, such as liver, kidney, heart or other organ meats or blood-based foods, including from wild game?	0 = No 1 = Yes	
C.	Any other types of meat or poultry, like beef, pork, lamb, goat, rabbit, wild game meat, chicken, duck, other birds?	0 = No 1 = Yes	
D.	Any edible insects, whether fresh or dried?	0 = No 1 = Yes	
E.	Any eggs; eggs from poultry or any other bird?	0 = No 1 = Yes	
F.	Any fish or seafood, whether fresh or dried?	0 = No 1 = Yes	
G.	Any beans or peas, such as mature beans or peas, lentils or bean/pea products, including *rupiza*?	0 = No 1 = Yes	
H.	Any nuts or seeds, like tree nut, groundnut/peanut, or certain seeds or nut/seed “butters” or pastes?	0 = No 1 = Yes	
I.	Any milk or milk products, such as milk, cheese, yoghurt or other milk products, but NOT including butter, ice cream, cream or sour cream?	0 = No 1 = Yes	
J.	Any oils/fat/butter?	0 = No 1 = Yes	
K.	Any condiments and seasonings, such as ingredients used in small quantities for flavour,	0 = No 1 = Yes	

	such as salt, chilies, spices, herbs, fish powder, tomato paste, flavour cubes or seeds?		
L.	Any other beverages and foods Tea or coffee if not sweetened, clear broth, alcohol?	0 = No 1 = Yes	

7: HOUSEHOLD ASSETS

[INTRODUCTION: Now I would like to ask you about some assets that your household currently own or not. I will go one by one and please let me know if your HH currently have the asset or not]

**N°**	**Questions**	**Responses**	**Code**
**Does your HH currently own this asset?**			
a.	Plough (oxen-pulled)	0 = No 1 = Yes	
b.	Scotch cart	0 = No 1 = Yes	
c.	Tractor (Mechanical plough)	0 = No 1 = Yes	
d.	Sickle	0 = No 1 = Yes	
e.	Pick axe	0 = No 1 = Yes	
f.	Axe	0 = No 1 = Yes	
g.	Pruning/cutting shears	0 = No 1 = Yes	
h.	Hoe	0 = No 1 = Yes	
i.	Spade or shovel	0 = No 1 = Yes	
j.	Traditional beehive	0 = No 1 = Yes	
k.	Modern beehive	0 = No 1 = Yes	
l.	Knapsack sprayer	0 = No 1 = Yes	
m.	Mechanical water pump	0 = No 1 = Yes	
n.	Motorized water pump	0 = No 1 = Yes	
o.	Stone grain mill	0 = No 1 = Yes	
p.	Motorized grain mill	0 = No 1 = Yes	
q.	Walking motorized tiller	0 = No 1 = Yes	
r.	Cultivator/ridger/planter	0 = No 1 = Yes	
s.	Sewing machine	0 = No 1 = Yes	
t.	Wheel barrow	0 = No 1 = Yes	
u.	Borehole	0 = No 1 = Yes	
v.	Well	0 = No 1 = Yes	
w.	Bicycle	0 = No 1 = Yes	
x.	Motorcycle	0 = No 1 = Yes	
y.	Vehicle	0 = No 1 = Yes	
z.	Mobile phone	0 = No 1 = Yes	
aa.	If mobile phone=yes	a) Kambudzi (Ordinary without many functions)<br>b) Smart phone	
bb.	Cattle	0 = No 1 = Yes	
cc.	If cattle =Yes how many does the household possess	0 = No 1 = Yes	

8: VALUE CHAIN OF EDIBLE INSECTS IN GENERAL

N°	Questions	Responses	Code
0	Does your household consume edible insects?	1. Yes<br>2. No	
8.1a	How many people in your household consume edible insects?		
8.1b	Who introduced you to consumption of edible insects?	1. Self interest<br>2. Family Tradition/Generational<br>3. Friends<br>4. Relatives	
8.2	Which age groups in your household consume insects? (*possible to give multiple responses)*<br>*To include the gender of the members [M/F]*	1. 6 – 23 months<br>2. 24 – 59 months<br>3. 6-9 years<br>4. 10 – 14 years<br>5. 15 – 19 years<br>6. 20-29 years<br>7. 30-39 years<br>8. 40-49 years<br>9. Above 50 years<br>Male<br>Female<br>Both	
8.3	Which edible insects do you consume? (*possible to give multiple responses)*	1. *Madora/Amacimbi/Gonimbrasia belina*<br>*2. Harati/Cerina forda*<br>*3. Magandari/Intowa/Inowa/Gonanisa maia*<br>*4. Majuru/Magenga/Macrotermes sp*<br>*5. Ishwa/inhlwa/Macrotermes*<br>*6. Mandere/Eulepida Mashona*<br>*7. Makurwe/Inyekevu/Brachytrupes membranaceous*<br>*8. Humbwe/Inyekevu/Acheta afer*<br>*9. Majenya/Henicus whellani*<br>*10. Tsambarafuta/Ihlabusi/Carebara vidua*<br>*11. Nyenze/Inyeza/Ioba leopardine*<br>*12. Harurwa/Umtshiphela/Encosternum delegorguei*<br>*13. Tsumwarumwa/Inswabanda/Ruspolia differens*<br>*14. Hwiza/Mhashu/Inthethe/ Locusta migratoria*<br>*15. Other*	
8.4a	Which edible insect is your most preferred?	1. *Madora/Amacimbi/Gonimbrasia belina*<br>*2. Harati/Cerina forda*<br>*3. Magandari/Intowa/Inowa/Gonanisa maia*<br>*4. Majuru/Magenga/Macrotermes sp*	

		5. Ishwa/inhlwa/Macrotermes<br>*6. Mandere/Eulepida Mashona*<br>*7. Makurwe/Inyekevu/Brachytrupes membranaceous*<br>*8. Humbwe/Inyekevu/Acheta afer*<br>*9. Majenya/Henicus whellani*<br>*10. Tsambarafuta/Ihlabusi/Carebara vidua*<br>*11. Nyenze/Inyeza/Ioba leopardine*<br>*12. Harurwa/Umtshiphela/Encosternum delegorguei*<br>*13. Tsumwarumwa/Inswabanda/Ruspolia differens*<br>*14. Hwiza/Mhashu/Inthethe/ Locusta migratoria*<br>*15. Other*	
8.5b	Reasons for preference (*possible to give multiple responses in order of importance)*	1. Relish<br>2. Taste<br>3. Nutritional value<br>4. Medicinal properties<br>5. Availability<br>6. Tradition/custom<br>7. Only food option (coping strategy)<br>8. Easy to harvest<br>9. Easy to process<br>10. Other (specify)	
8.6a	Do you or your household have any knowledge on nutritional value (benefits) of edible insects?	1. Yes<br>2. No	
8.6b	What is source of this information? (*possible to give multiple responses in order of importance)*	1. Literature/acquired knowledge<br>2. Extension workers<br>3. Friends<br>4. Relatives<br>5. Social gatherings<br>6. Radio<br>7. TV,<br>8. Print media (e.g., Newspaper)<br>9. Social media<br>10. Other (specify)	

9: MOPANE WORM VALUE CHAIN

N°	Questions	Responses	Code
9.0	Are you/any household member involved in the mopane worm value chain this includes (harvesting, processing, trading and consumption)	1. Yes<br>2. No	
9.1	Which actor(s) in the mopane value chain are you? (multiple response possible)	1. Harvester<br>2. Processor<br>3. Trader<br>4. Consumer	
9.2a	How many people in your household consume mopane worms?		
9.2b	Who introduced you to consumption of mopane worms?	1. Self interest<br>2. Family Tradition/Generational<br>3. Friends<br>4. Relatives<br>5. Other(specify)	
9.2c	Has anyone in your household reacted (allergic reaction) after consuming mopane worms?	1. Yes<br>2. No	
9.2d	What symptoms did they show (multiple answers possible). Question asked to those who replied yes to question 9.2c	1. Itchy skin<br>2. Running stomach<br>3. Vomiting<br>4. Tingling in the month<br>5. Swelling<br>6. Other (specify)	
9.3	How often do you consume mopane worms when in season?	1. Daily<br>2. ≥ 3 days per week<br>3. 1-2 days a week<br>4. Once a month<br>5. Does not consume	
9.4	What are the reasons for your household consuming mopane worms (*possible to give multiple responses*)	1. Relish<br>2. Taste<br>3. Nutritional value<br>4. Medicinal properties<br>5. Availability<br>6. Tradition/custom<br>7. Only source of food/ coping strategy<br>8. Easy to harvest<br>9. Easy to process<br>10. Other (specify)	
9.5	In which form does your household consume mopane worm? fresh or dried?	1. Fresh<br>2. Dried	

		3. Both	
9.6a	Do you/ your household member harvest mopane worms?	1. Yes<br>2. No	
9.6b	Do you/ your household member harvest mopane worms for household consumption or for trade?	1. Only for household consumption<br>2. Only for trade<br>3. For both	
9.7	Who is involved in harvesting mopane worms in your household?	1. Mother only<br>2. Father only<br>3. Children only<br>4. Mother and father<br>5. Mother and children<br>6. Father and children<br>7. Whole family<br>8. Any other household member	
9.8.	Where do you harvest mopane worms? (Multiple answers possible)	1. Within the ward<br>2. Outside the ward but within nearby wards<br>3. Outside the ward at distant wards	
9.9.	What time of the day do you harvest mopane worms?	1. Morning<br>2. Afternoon<br>3. Evening<br>4. Anytime of the day	
9.10	In which season do you harvest most of the mopane worms?	1. October -December season<br>2. February-May season	
9.11a	How much do you normally harvest per season (If replied 1 for question 9.1)	October-December season | | Quantity 1. Kg 2. 5 litre tin 3. 20 litre tin 4. 50 kg bag | Unit | <br>Feb-May season | Quantity 1. Kg 2. 5litre tin 3. 20litre tin 4. 50kg bag | Unit	
9.11b	How much of the harvested quantities did you consume?	October-December season | | Quantity 1. Kg 2. 5 litre tin 3. 20 litre tin 4. 50 kg bag | Unit | <br>Feb-May season | Quantity | Unit	

		1. Kg<br>2. 5litre tin<br>3. 20litre tin<br>4. 50kg bag	
9.11c	How much of the harvested quantities do you sell?	October-December season<br>Quantity: 1. Kg 2. 5 litre tin 3. 20 litre tin 4. 50 kg bag<br>Unit<br><br>Feb-May season<br>Quantity: 1. Kg 2. 5litre tin 3. 20litre tin 4. 50kg bag<br>Unit	
9.11d.	What is estimated total income generated by the household from the quantities collected (RTGS/USD/Rand/Pula)	October-December season / Feb-May season	
9.12	Whom do you sell your mopane worms to?	1. Other households in the area<br>2. Informal traders (middleman)<br>3. Informal markets (roadside and open markets)<br>4. Formal markets (retail)<br>5. Export market<br>6. Barter trading<br>7. Other (specify)	
